# The relationship between postpartum care uptake and postpartum morbidity and their determinants in Morocco: a secondary analysis of a national survey

**DOI:** 10.1186/s12889-026-26774-x

**Published:** 2026-04-09

**Authors:** Asmaa Habib, Hafiz T. A. Khan, Caroline Lafarge, Rachid Bezad

**Affiliations:** 1 Faculty of Medical Sciences, UM6P Hospitals, VI Polytechnic University, Benguerir, 43150 Morocco; 2 Public Health Group, University of West London, Brentford, Middlesex TW8 9GB UK; 3 Oxford Institute of Population Ageing, University of Oxford, Oxford, OX2 6PR UK; 4 Graduate School, University of West London, St Mary’s Road, London, W5 5RF UK; 5 Faculty of Medicine and Pharmacy, University Mohamed V, Rabat, Morocco

**Keywords:** Postpartum care, Postpartum morbidity, Maternal health, Health inequities, Morocco

## Abstract

**Background:**

Postpartum care (PPC) utilisation is essential to prevent maternal mortality and morbidity, particularly in low-and middle-income countries where 95% of maternal deaths occur. In Morocco, PPC remain underused. This study examines sociodemographic, environmental and obstetric factors associated with PPC utilisation and postpartum morbidity (PPM), and the relationship between PPC and PPM.

**Methods:**

A secondary data analysis of a nationally representative dataset of 5,593 women of childbearing age was conducted. Univariate and multivariate logistic regression assessed the associations between determinants and PPC utilisation and PPM.

**Findings:**

About 53.6% of women received PPC consultation before discharge, 21.8% within 6 weeks post-delivery (later PPC, LPPC) and 28.3% reported PPM. Determinants positively associated with PPC utilisation included women’s education above primary level (AOR = 1.34, 95% CI:1.11–1.63), high household wealth index (AOR = 1.42, 95%CI:1.02–1.98), antenatal care (AOR = 1.64, 95% CI:1.08–2.47), caesarean delivery (AOR = 2.50, 95%CI:1.89–3.31), and newborn postnatal care (AOR = 6.97, 95% CI:5.89–8.25). Absence of doctors during midwives-led delivery reduced LPPC uptake (AOR = 0.63, 95% CI:0.48–0.83). Secondary or higher education (AOR = 0.71, 95% CI:0.54–0.93) and antenatal care (AOR = 0.30, 95% CI:0.14–0.65) reduced PPM risk, while instrumental delivery (AOR = 1.24, 95% CI:1.04–1.48) and pregnancy morbidities (AOR = 2.10, 95% CI:1.72–2.56) increased it. Early PPC (EPPC) provision during hospitalisation lowered PPM risk (AOR = 0.65, 95% CI:0.52–0.79), whereas LPPC utilisation was associated with PPM occurrence (AOR = 1.36, 95% CI:1.08–1.71).

**Conclusion:**

PPC utilisation remains low, even during delivery-led hospitalisation, and PPM persists, reflecting health inequities. Interventions toward women with low sociodemographic characteristics are needed to increase PPC utilisation and prevent PPM. Further qualitative research is essential to explore behavioral and cultural influences and to address women’s and health professionals’ perceptions of PPC.

**Supplementary Information:**

The online version contains supplementary material available at 10.1186/s12889-026-26774-x.

## Background

The World Health Organisation (WHO) recognises the importance of using postpartum care (PPC) consultations to screen and raise awareness of symptoms of the most common postpartum morbidities (PPM) and inform women about good postpartum practices. Since 2013, they recommend the uptake of four PPC follow-ups: the first during the 24 h after delivery- provided before discharge for delivery-led hospitalisation, the second 72 h post-delivery, the third between the seventh and the 14th day post-delivery, and the fourth six weeks after delivery [1]. The first two consultations should happen during the acute postpartum period (the first week after delivery) when sudden severe metabolic complications can occur, and the two others should take place during the second phase of the postpartum period (between the second and sixth weeks after delivery). Globally, 71% of women received a PPC check-up within 48 h after delivery [2]. The uptake of PPC consultations by women is influenced by several sociodemographic and obstetric determinants, especially in low-and-middle income countries (LMIC). In low-resource settings, living in rural areas, transportation, poverty, illiteracy, unemployment, single marital status, lack of autonomy in decision-making, mistreatment by health professionals during maternal care and some specific cultural beliefs such as seclusion can impede PPC uptake, whereas attendance to antenatal care visits, skilled-birth delivery, caesarean delivery and primiparity contribute to PPC provision [3, 4].

Postpartum care (PPC) utilisation is essential to prevent postpartum maternal mortality and morbidity, particularly in LMIC where 95% of maternal death occur [5]. Haemorrhage, indirect obstetric deaths and hypertensive disorders (eclampsia) were the most life-threatening conditions and were responsible for over half of maternal deaths in the world [6–8]. More than two thirds of haemorrhage-related deaths occurred within six weeks after childbirth [7]. Maternal mortality in Morocco has declined substantially (35%) from 112 to 72.6 deaths per 100,000 live births between 2010 and 2018 [9, 10]. Although this progress is encouraging, the maternal mortality ratio (MMR) remains slightly above the Sustainable Development Goal 3 (under 70 deaths per 100,000 live births), and marked with socio-demographic disparities. For example, in 2018, the MMR gap between urban and rural areas was large: 44.5 versus 111.1 deaths per 100,000 live births respectively [9]. Additionally, despite free delivery care in public facilities, out-of-pocket payments for medicines reaching around 200 dirhams (approximately 8% of the average monthly income in 2017) remain common and may constitute a significant barrier to accessing PPC [11]. Therefore, women’s socio-demographic status appears to be an important factor in maternal health inequalities [12].

In 2011, only 21.8% of women living in Morrocco reported attending PPC consultations [13]. PPC are provided by midwives, gynecologists or general practitioners in both public and private sectors. The mandatory hospitalisation stay after delivery is 24 h during which at least one early PPC (EPPC) check-up should be delivered either in public hospitals maternity wards, delivery centres or in private clinics. Later PPC (LPPC) consultations should occur after discharge within six weeks postpartum, free of charge in all public delivery centres or at a cost in private surgeries. In Moroccan settings, research on PPM started in 2005 [14] but few studies have since investigated this topic. In the latter, the complications reported by women included haemorrhage (79.9%), fever (12.1%), pregnancy-related hypertension (10.6%), mental disorders (10.0%), genital infections (8.0%), and breast conditions (5.0%) [15, 16]. The literature showed that several factors are positively associated with PPC uptake including women’s education, middle or high household wealth index, urban place of residence, attendance to antenatal care visits, health facility based-delivery, caesarean delivery, skilled-birth deliveries, women’s autonomy in decision making [3, 17, 4].

The aim of this study was, to determine the scope of PPC uptake in Morocco and understand its barriers and facilitators determinants. The second objective was to assess the relationships between PPC uptake and PPM occurrence in Morocco.

## Methods and materials

### Study design and population

**Fig. 1:**
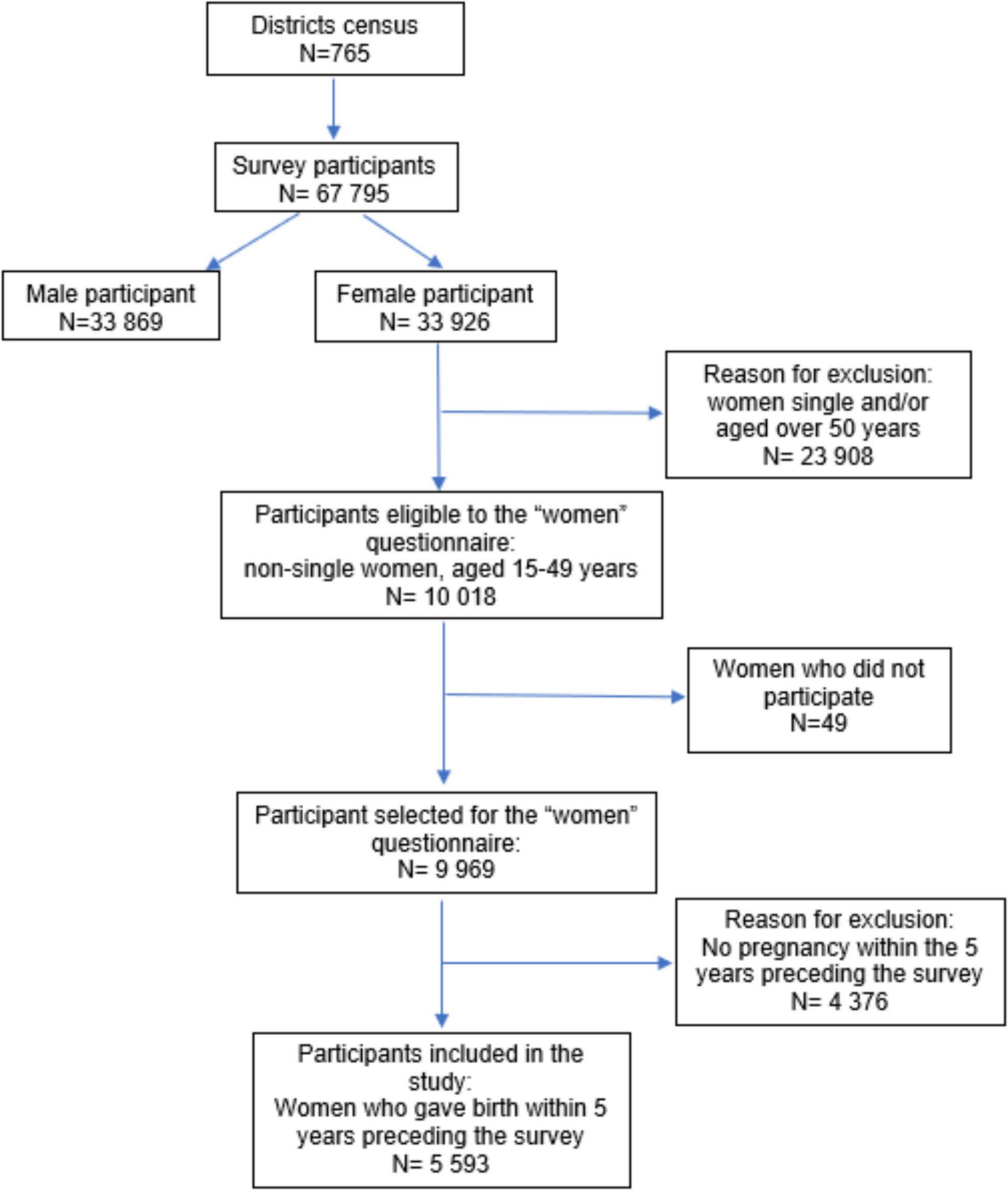
Flowchart of the selection process of the study participants

The study was based on secondary data analysis of the women’s health questionnaire (Sects. 1, 3 and 5) of the *Enquête Nationale sur la Population et la Santé Famililale* (ENPSF)- National Survey of Population and Family Health 2018 which is a large-scale cross-sectional survey used in Morocco [18]. The questionnaire is constructed from the Multiple Indicator Cluster Surveys and the Pan Arab Project for Family Health questionnaires and adapted to the Moroccan context. Figure 1 presents the study selection process of participants. The sampling methods used a two-stage stratified probability sampling method ensuring the representativeness of the data to the total population of the country. In the first stage, a probabilistic sample of census districts was selected within each sampling stratum, taking into account the varying levels of representativeness of the results. In the second stage, a probabilistic sample of households was drawn from each selected census district. Survey weights were applied to ensure representativeness [18]. More details are available in the ENPSF 2018 report (p.190–212) [18]. Eligible participants were women aged between 15 and 49 years, who gave birth to a live baby within five years prior to the survey (from 2013 to 2017). For women with more than one live birth during this period, the questionnaire focused on the most recent live birth. Eligible women were non-single (meaning married, divorced, separated, or widowed) at the time of the survey (Fig. 1).

### Outcome variables

Three dependent variables were analysed. The first one was ‘early PPC uptake’ (EPPC) before discharge from hospital. Analyses related to this dependent variable excluded women who delivered at home or other places. The second variable was ‘later PPC uptake’ (LPPC) within six weeks post-delivery. The third variable was PPM occurrence, within six weeks after delivery, which included the following physical health complications: acute vaginal haemorrhage, oedema and foot pain, smelly vaginal discharge with fever, pelvic pain with fever, lower back pain with fever, dorsal pain with fever, urinary burning with fever, pain and swelling mammary with fever, and other morbidities that were not defined in the database. All the outcome variables were binary (Yes/No). More detail about additional characteristic of outcome variables are available in the Supplementary file- Table 1.

### Independent variables

Altogether, 55 predictors (or independent variables) were analysed and classified into four main categories: sociodemographic (eight variables), environmental (four variables), obstetric (27 variables) and ‘other’ (16 variables). In this article, only predictors reported in the literature and those included in the multivariate analyses as covariates are presented. Among the obstetric predictors, the pregnancy health complications variable considered abnormal swelling of the face, fingers, and feet; vaginal haemorrhage; convulsion not caused by fever; intense and persistent headache; blurry vision; intense pelvic pain; hyperventilation; fever with difficulty standing up; and water break six hours before labour. Moreover, the variables postnatal care (PNC) before discharge and PNC within six weeks post-delivery refers to the consultations provided to the newborn baby. More details about all independent variables are provided in the Women health questionnaire available in the National Survey on Population and Family Health 2018 report (p.226–231, p.235–240) [18].

### Statistical analysis

Two of the 55 predictors had entries with missing data, which were removed prior to analysis. To describe the population of study, the distributions of dependent and independent variables were assessed. Moreover, bivariate analyses were performed to estimate the associations between predictors and EPPC, LPPC and PPM, independently. For predictors with two modalities, Chi-squared tests were carried out, whereas bivariate analyses were chosen to examine the associations with predictors defined by at least three categories. The effect sizes were expressed by crude odds ratios with a 5% significance level.

Secondly, a multivariate regression was conducted independently for each of the three outcome variables, which enabled their inclusion as independent variables in the models. We considered a diagnostic test of multicollinearity (i.e., variance inflation factor) to determine the predictors for inclusion in the logistic regression (cf. supplementary file- Table 2 and Table 3). Only predictors that did not induce serious multicollinearity [19] and already used in the literature [4, 17] were deemed eligible to generate reliable statistical regression models. A hierarchical multiple regression method with two models specified for each outcome was used. Model 1 included only sociodemographic and environmental predictors, and model 2 accounted for model 1 plus obstetric predictors. The results were reported as adjusted odds ratios, estimated with a 5% level of statistical significance. The analyses were performed using the Statistical Package for the Social Sciences software IBM SPSS V28 [20].

## Results

### Characteristics of respondents

Altogether, 5,593 women were included in this study (Fig. 1). Analyses related to EPPC applied to women who delivered in a health facility, accounting for 4,792 women.

Participants’ mean age was 31.7 (SD:6.8), most women were married (97.5%), unemployed (90.0%), and without formal education (57.8%) or educated to a primary level (32.8%). Only few (9.4%) reached secondary and higher education (Table 1).

**Table 1:** Distribution of women’s characteristics (*N* = 5,593, except for early postpartum care with *n* = 4,792)

Characteristics	Frequency (n)	Percent (%)
Postpartum care uptake before discharge from health facility (EPPC)		
No	1792	32.0
Yes	3000	53.6
Postpartum care uptake within 6 weeks post-delivery (LPPC)		
No	4372	78.2
Yes	1219	21.8
LPPC location		
Public hospitals	160	13.1
Public health centres with and without delivery unit	487	40.0
Private clinics	187	15.4
Private surgeries	368	30.2
Home	15	1.2
Postpartum morbidity (PPM)		
No	4010	71.7
Yes	1583	28.3
Frequency of PPM per woman		
No morbidities	4010	71.1
1 morbidity	879	15.7
2 morbidities	311	5.6
3 morbidities	149	2.7
4 morbidities	107	1.9
5 morbidities	67	1.2
6 morbidities	35	0.6
7 morbidities	23	0.4
8 morbidities	8	0.1
9 morbidities	4	0.1
Postpartum pelvic pain with fever		
No	5162	92.3
Yes	426	7.6
Do not know	4	0.1
Other PPM		
No	5356	95.8
Yes	237	4.2
Maternal age		
15–29	2289	40.9
30–39	2520	45.1
40–49	783	14.0
Maternal education		
No formal education	3235	57.8
Primary education	1835	32.8
Secondary/higher education	523	9.4
Partner education level		
Primary	1851	44.0
Preliminary/Moderate	1133	27.0
Secondary/Higher	1220	29.0
Maternal employment status		
Unemployed	5035	90.0
Employed	558	10.0
Household wealth index		
Poorest	1275	22.8
Poorer	1146	20.5
Middle	1167	20.9
Richer	1128	20.2
Richest	876	15.7
Marital status		
Married	5452	97.5
Widow	41	0.7
Divorced	72	1.3
Separated	28	0.5
Place of residence		
Urban	3128	55.9
Rural	2464	44.1
Regions		
Tanger-Tétouan- Al Hoceima	642	11.5
Oriental	389	7.0
Fès- Meknès	706	12.6
Rabat—Salé -Kénitra	675	12.1
Béni Mellal -Khénifra	391	7.0
Casablanca- Settat	1031	18.4
Marrakech-Safi	869	15.5
Drâa-Tafilalet	282	5.0
Souss-Massa	446	8.0
Guelmim-Oued Noun	65	1.2
Laâyoune – Sakia El Hamra	73	1.3
Dakhla – Oued ed Dahab	23	0.4
Long distance from a health facility preventing LPPC uptake		
Yes	122	2.8
No	4251	97.2
Antenatal care		
0 visit	701	12.5
1 to 3 visit(s)	1926	34.4
4 visits	1207	21.6
> 4 visits	1759	31.4
Last antenatal care location		
Public hospital	275	5.6
Health centre/delivery centres	1809	36.8
Private clinic	200	4.1
Private surgery	2618	53.3
Home	9	0.2
Number of morbidities during pregnancy		
No morbidities	3092	55.3
1 morbidity	1041	18.6
2 morbidities	542	9.7
3 morbidities	378	6.8
4 morbidities	246	4.4
5 morbidities	160	2.9
6 morbidities	82	1.5
7 morbidities	33	0.6
8 morbidities	18	0.3
9 morbidities	2	0.0
Mode of delivery		
Vaginal delivery	1835	38.2
Vaginal delivery assisted by instruments	1966	40.9
Caesarean delivery	1008	21.0
Birth attendant		
Doctors	1053	21.9
Nurses/Midwives	3051	63.4
Doctors + Nurses/Midwives	707	14.7
Place of delivery		
Home	770	13.8
Public hospital	3162	56.8
Delivery centre/health centre	760	13.7
Private clinic	811	14.6
Private surgery	62	1.1
Length of stay in the health facility after birth		
< a day (hours)	461	9.6
1 ≥ days ≤ 7	4278	89.3
≥ 1 week	53	1.1
Postnatal care before discharge		
No	1536	32.2
Yes	3239	67.8
Postnatal care within six weeks		
No	3601	64.5
Yes	1981	35.5
Contraception usage		
No	654	12.9
Yes	4404	87.1

### Utilisation of postpartum care and postpartum morbidity prevalence

About 53.6% of women received EPPC during delivery-led hospitalisation, but only 21.3% used LPPC within six weeks post-delivery. LPPC were essentially taken in public health facilities or delivery centres (40.0%), and in private surgeries (30.2%) (Table 1). Conversely, 78.2% of women did not use LPPC. The main reasons included the absence of PPM (70.6%), lack of awareness of PPC importance (15.2%), financial difficulty (7.5%), distance to health facility (2.8%), unavailability of LPPC services (1.7%), and other unspecified reasons.

Overall, 28.3% of women reported at least one PPM, with most reporting only a single symptom (15.7%) (Table 1).

### Factors associated with early postpartum care uptake

The findings regarding EPPC uptake are displayed in Table 2.

**Table 2:** Multilevel logistic regression of factors associated with early postpartum care uptake

Variables	EPPC	EPPC	OR (95% CI)	Adjusted OR	
	No (%)	Yes (%)		Model 1	Model 2
Maternal age					
15-29	41.4	58.6	1	1	1
30-39	35.4	64.6	1.29 (1.14-1.46)***	1.26 (1.10-1.44)***	1.16 (0.96-1.39)
40-49	31.7	68.3	1.52 (1.26-1.83)***	1.49 (1.23-1.81)***	1.24 (0.95-1.64)
Maternal education					
No formal education	41.1	58.9	1	1	1
Primary	36.3	63.7	1.22 (1.08-1.39)**	1.26 (1.10-1.44)***	1.06 (0.88-1.29)
Secondary and higher	22.7	77.3	2.37 (1.90-2.95)***	1.93 (1.52-2.45)***	1.23 (0.88-1.73)
Partner’s education level					
Primary	43.0	57.0	0.40 (0.34-0.47)**		
Preliminary/Moderate	39.2	60.8	0.47 (0.39-0.56)**		
Secondary and higher	23.2	76.8	1		
Women’s employment					
Unemployed	38.7	61.3	1	1	1
Employed	26.4	73.6	1.76 (1.43-2.16)***	1.33 (1.07-1.66)**	1.14 (0.84-1.53)
Household wealth index					
Poorest	39.3	60.7	1	1	1
Poorer	43.6	56.4	0.84 (0.69-1.01)	0.75 (0.62-0.91)**	0.85 (0.65-1.11)
Middle	39.8	60.2	0.98 (0.81-1.18)	0.77 (0.62-0.95)*	0.83 (0.62-1.11)
Richer	34.6	65.4	1.23 (1.02-1.48)*	0.87 (0.69-1.09)	0.86 (0.63-1.19)
Richest	29.1	70.9	1.58 (1.29-1.93)***	1.03 (0.80-1.33)	0.85 (0.60-1.20)
Place of residence					
Rural	43.1	56.9	1	1	1
Urban	33.9	66.1	1.47 (1.31-1.66)***	1.23 (1.04-1.45)*	0.98 (0.77-1.23)
Antenatal care					
0 visit	46.9	53.1	1		1
1 to 3 visit(s)	42.7	57.3	1.18 (0.94-1.49)		1.04 (0.75-1.44)
4 visits	38.1	61.9	1.44 (1.13-1.82)**		1.00 (0.71-1.41)
> 4 visits	29.7	70.3	2.09 (1.66-2.64)***		1.08 (0.77-1.51)
Mode of delivery					
Vaginal delivery	41.4	58.6	1		1
Vaginal delivery assisted by instruments	45.5	54.5	0.85 (0.75-0.96)*		0.85 (0.71-1.02)
Caesarean delivery	14.4	85.6	4.18 (3.43-5.11)***		2.60 (1.86-3.65)***
Birth attendant					
Doctors	19.3	80.7	1		1
Nurses/Midwives	46.1	53.9	0.28 (0.24-0.33)***		0.84 (0.63-1.12)
Doctors+Nurses/Midwives	26.8	73.2	0.65 (0.52-0.82)***		1.13 (0.82-1.55)
Postnatal care before discharge					
No	84.5	15.5	1		1
Yes	15.0	85.0	30.88 (26.09-36.55)***		27.91 (23.29-33.45)***
Postnatal care within six weeks					
No	43.7	56.3	1		1
Yes	26.3	73.7	2.17 (1.91-2.47)***		1.13 (0.93-1.36)
Number of morbidities during pregnancy					
No morbidity	34.8	65.2	1		1
1 morbidity	36.5	63.5	0.93 (0.79-1.09)		0.80 (0.65-1.00)
2 morbidities	43.0	57.0	0.71 (0.58-0.86)***		1.07 (0.79-1.44)
3 morbidities	43.7	56.3	0.69 (0.55-0.87)**		0.89 (0.63-1.27)
4 morbidities	44.7	55.3	0.66 (0.50-0.87)**		1.00 (0.65-1.52)
5 morbidities	36.3	63.7	0.94 (0.66-1.33)		1.22 (0.72-2.07)
6 morbidities	58.0	42.0	0.39 (0.24-0.63)***		0.75 (0.37-1.55)
7 morbidities	41.4	58.6	0.76 (0.36-1.61)		1.25 (0.39-3.97)
8 morbidities	34.1	65.9	1.03 (0.36-2.98)		2.87 (0.71-11.54)
9 morbidities	33.3	66.7	1.07 (0.05-24.98)		
Place of delivery					
Public hospital	44.0	56.0	1		
Delivery centre or health centre	37.2	62.8	1.32 (1.13-1.56)**		
Private clinic	14.2	85.8	4.75 (3.85-5.85)**		
Private surgery	8.7	91.3	8.24 (3.38-20.12)**		
Length of stay in a health facility post-delivery					
< a day (hours)	47.1	52.9	1		
1≥ days ≤ 7	36.5	63.5	1.54 (1.27-1.87)**		
≥ week	22.2	77.8	3.12 (1.59-6.13)**		

Model 1: adjusted for sociodemographic and environmental variables

Model 2: adjusted for all variables

*EPPC* Early postpartum care

*OR* Odds ratios

*p<0.05

**p<0.01

***p<0.001

Maternal sociodemographic characteristics associated with EPPC in Morocco were being aged 30–49 years old, having completed formal education (regardless of the level for women and at least of secondary for their partner), being employed, and living in richer or the richest households. However, the logistic regression (Table 2) suggests that when taken into consideration all predictors alongside obstetric factors (model 2), sociodemographic factors were no longer significant predictors of EPPC.

Similarly, urban place of residence, attending at least four antenatal care visits and experiencing health complications during pregnancy were additional predictors initially found to facilitate EPPC uptake, but this was not confirmed by the multivariate analysis (Table 2-model 2).

In these analyses (Table 2-model 2), delivery by caesarean section was significantly associated with EPPC. Women who delivered by a caesarean section were 2.6 times more likely to receive EPPC compared to their counterparts who had a straight vaginal delivery. Newborn postnatal care (PNC) before discharge was another significant predictor of EPPC uptake as it multiplied by 27.9 times the likelihood of women receiving EPPC for themselves. Thus, the PNC is an opportunity for health professionals to examine both women and babies at the same time.

### Factors associated with later postpartum care uptake

Findings regarding LPPC uptake are displayed in the supplementary file- Table 4.

Age, higher level of education and household wealth index were all significantly associated with LPPC. Regarding the age predictor, women aged 30 to 39 were 23% (AOR = 1.23, 95%CI:1.02–1.48) more likely to use LPPC compared to younger women (aged 15 to 29). Besides, compared to no formal education, primary level and secondary or higher education level increased by 34% (AOR = 1.34, 95%CI:1.11–1.63) and 79% (AOR = 1.79, 95%CI:1.35–2.36) respectively, the likelihood of using LPPC. Therefore, the higher the level of education the higher likelihood of LPPC uptake. Similarly, belonging to rich and richest household increased by 42% (AOR = 1.42, 95%CI:1.02–1.98) and 66% (AOR = 1.66, 95%CI:1.18–2.34) the odds of PPC utilisation, respectively.

Furthermore, compared to non-attendance to ANC consultations, the likelihood of LPPC uptake increased by 64% (AOR = 1.64, 95%CI:1.08–2.47), 88% (AOR = 1.88, 95%CI:1.23–2.86), and 89% (OR = 1.89, 95%CI:1.25–2.86) for women who received one to three ANC visits, four visits, and more than four visits, respectively. Therefore, the higher the frequency of ANC check-ups, the higher the likelihood of using LPPC.

The type of health professionals who assisted the delivery also had a significant association with LPPC uptake. The presence of only midwives or nurses decreased by 37% (AOR = 0.63, 95%CI:0.48–0.83) the odds of LPPC uptake compared to a delivery assisted by a doctor. Additionally, women who delivered through caesarean were 2.50 times (AOR = 2.50, 95%CI:1.89–3.31) more likely to use LPPC than their counterparts who gave birth through non-instrumental vaginal delivery.

Another facilitator of LPPC was the provision of PNC to newborn babies within the six weeks post-delivery. Women were almost seven times (AOR = 6.97, 95%CI:5.89–8.25) more likely to use LPPC when their babies received PNC within the same period (supplementary file- Table 4).

### Factors associated with postpartum morbidity

The findings regarding PPM occurrence are displayed in the supplementary file-Table 5.

Among the sociodemographic predictors of PPM, education level was significantly associated with PPM occurrence within six weeks post-delivery. Achieving secondary or higher the level of education, decreased the risk of developing PPM by 29% (AOR = 0.71, 95%CI:0.54–0.93). Regarding the obstetric predictors analysed, some appeared to have a protective influence, namely receiving ANC (AOR = 0.23, 95%CI:0.11–0.50), whilst others were identified as risk factors of PPM, such as the mode of delivery (i.e. instrumental vaginal delivery) (AOR = 1.24, 95%CI:1.04–1.48). Moreover, women who suffered from at least one health issue during pregnancy were significantly, from 2 (AOR = 2.10, 95%CI:1.72–2.56) to 15 (AOR = 15.33, 95%CI:5.43–43.26) times, more likely to experience PPM. Consequently, the higher the frequency of pregnancy morbidities, the higher the likelihood of PPM occurrence (supplementary file—Table 5).

### The relationship between early postpartum care (EPPC), later postpartum care (LPPC) and postpartum morbidtity (PPM)

**Fig. 2:**
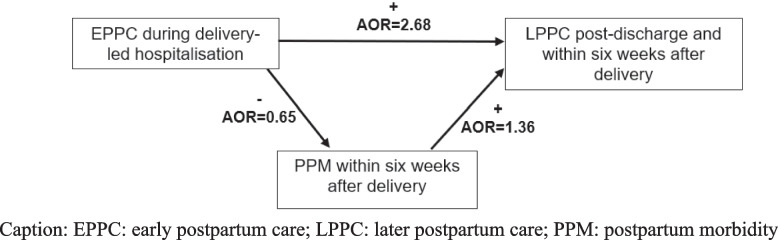
Framework illustrating the relationship between early postpartum care provision, later postpartum care uptake and postpartum morbidity. Caption: EPPC: early postpartum care; LPPC: later postpartum care; PPM: postpartum morbidity

The relationship between PPC and PPM, displayed in Table 3 and Fig. 2, indicates that receiving EPPC (before discharge) increased by 2.6 times the likelihood of LPPC uptake (within 6 weeks) and decreased the risk of PPM occurrence by 35%. Thus, EPPC is associated with lower odds of PPM occurrence at a later stage. In fact, women who reported one, three, four, and seven PPM were 23%, 69%, 77%, and 96% less likely to have received EPPC respectively (Table 3). These observations underline the importance of medical assistance during delivery and the following days.

Furthermore, suffering from one PPM was significantly associated with a 36% increased likelihood to receive LPPC (Table 3). Therefore, women seem to consider LPPC check-ups as a curative approach and use it to seek medical assistance if they experience PPM.

**Table 3:** Associations between early postpartum care, later postpartum care and postpartum morbidity

Variables	EPPC				LPPC				PPM			
	No %	Yes %	OR (95%CI)	Model 2 – Adjusted OR (95%CI)	No%	Yes%	OR (95%CI)	Model 2-Adjusted OR (95%CI)	No%	Yes%	OR (95%CI)	Model 2-Adjusted OR (95%CI)
EPPC												
No					91.0	9.0	1	1	66.8	33.2	1	1
Yes					68.7	31.3	4.61 (3.85–5.52)***	2.68 (2.08–3.45)***	74.8	25.2	0.68 (0.60–0.77)***	0.65 (0.52–0.79)***
LPPC												
No									73.2	26.8	1	1
Yes									66.2	33.8	1.39 (1.22–1.60)***	1.76 (1.46–2.13)***
PPM												
No morbidity	34.8	65.2	1	1	79.9	20.1	1	1				
1 morbidity	40.5	59.5	0.78 (0.67–0.92)**	0.77 (0.61–0.97)*	73.1	26.9	1.46 (1.23–1.73)***	1.36 (1.08–1.71)**				
2 morbidities	39.3	60.7	0.83 (0.64–1.07)	0.91 (0.62–1.34)	73.1	26.9	1.46 (1.12–1.90)**	1.24 (0.86–1.81)				
3 morbidities	57.5	42.5	0.39 (0.28–0.56)***	0.31 (0.19–0.50)***	76.0	24.0	1.25 (0.85–1.84)	1.14 (0.64–2.03)				
4 morbidities	58.9	41.1	0.37 (0.24–0.57)***	0.23 (0.12–0.45)***	75.4	24.6	1.30 (0.83–2.03)	1.19 (0.59–2.40)				
5 morbidities	50.6	49.4	0.52 (0.30–0.90)*	0.71 (0.32–1.60)	78.0	22.0	1.12 (0.62–2.00)	0.38 (0.16–0.95)*				
6 morbidities	49.7	50.3	0.54 (0.26–1.13)	0.48 (0.19–1.25)	77.9	22.1	1.12 (0.50–2.50)	0.93 (0.30–2.91)				
7 morbidities	80.7	19.3	0.13 (0.03–0.51)**	0.04 (0.01–0.23)***	84.0	16.0	0.76 (0.25–2.30)	0.29 (0.04–2.41)				
8 morbidities	32.5	67.5	1.11 (0.17–7.37)	0.35 (0.04–3.03)	61.5	38.5	2.48 (0.61–10.13)	3.20 (0.32–31.96)				
9 morbidities	37.9	62.1	0.88 (0.10–7.58)	0.20 (0.02–2.37)								

Model 2: adjusted for all variables (sociodemographic, environmental, obstetrical covariates)

*EPPC* Early postpartum care, *LPPC* Later postpartum care, *PPM* Postpartum morbidity

*OR* Odds ratios

^*^p < 0.05

^**^p < 0.01

^***^p < 0.001

## Discussion

The extent of PPC utilisation in Morocco reached 53.6% before discharge and 21.8% within six weeks of delivery. Thus, the prevalence of LPPC utilisation stagnated between 2011 and 2018 [13]. Moreover, 28.3% of PPM (pregnancy-related morbidities) within six weeks post-delivery were reported. Several sociodemographic factors influenced positively later PPC uptake in Morocco as in other LMIC [4, 17], including: being over 30 years old, achieving formal education, and belonging to wealthier households. Women with higher household wealth index were more likely to use LPPC, as their financial means enable them to afford continuous monitoring throughout the maternity process in the private sector by the same health professional. This may foster trustful and continuous relationships post-discharge. Education appeared particularly beneficial too, with lower risk of developing PPM and higher odds of LPPC uptake, it contributes to women’s knowledge and awareness of PPC benefits, leading to proactive health seeking-behaviours [21, 22]. However, this study (cf. Model 2), did not find significant associations between women’s employment status or place of residence and LPPC uptake, diverging from findings in others LMIC [23, 24].

Among obstetric determinants, the mode of delivery influenced PPC uptake. Caesarean deliveries doubled EPPC and LPPC utilisation, which was similar to the rate reported by other LMIC [23]. This may be due to required post-surgical follow-up which might be assimilated to EPPC [25], whereas vaginal delivery, perceived as a natural process, may not prompt the same level of follow-up, especially in heavy workload situations, unless complications arise. Instrumental vaginal delivery was also associated with an increased risk of PPM, consistent with findings from other LMIC reported in the systematic review by Sobhy et al. [26]. Procedures such as episiotomy, which is a surgical incision of the perineum to prevent perineal tears often performed during instrumental delivery [27], can increase the risk of puerperal infection; like inappropriate and insufficient self-care practice [28, 29]. Nevertheless, strengthening training and education of health professionals could enhance the efficiency of resource use and improve women's postpartum health [30].

The results also showed that PNC provided to newborn babies was the strongest predictor of both EPPC and LPPC. Thus, whether in hospital or within six weeks post-delivery, the care given to babies, such as vaccination, was related to the care provided to women.

The only identified barrier to LPPC uptake was being assisted through delivery by midwives/nurses but without a doctor’s presence. In Morocco, most deliveries occur in public hospitals and are midwife-led for uncomplicated vaginal birth without the assistance of doctors, in line with national regulations [31]. However, antenatal and postpartum consultations often delivered by doctors or midwives/nurses are mainly provided in other types of health facilities. In this context, childbirth is an event that generally only involves a short and temporary relationship between women and health professionals. The latter may perceive their responsibilities as limited to care provided within hospital settings, weakening the continuity needed to promote follow-up. Therefore, strengthening maternal referral pathways in public health facilities is needed, especially after delivery, to ensure continuity of care. Conversely, in private sector, a single health professional often provides care across the maternal health continuum from ANC to LPPC. Their duty of care focuses on the woman rather than the facility, which encourage them promoting LPPC consultations. Besides, giving birth in a health facility, in particular private structures, also appeared as a potential facilitator of EPPC and LPPC uptake (cf. bivariate analysis), a trend also observed in Zambia [32]. These results emphasise the importance of the health professional-woman relationship in ensuring continuity of care [33].

Furthermore, the results indicated that attending four ANC consultations was positively associated with EPPC and LPPC uptake, and reduced PPM risk, underscoring the important role of antenatal counselling in promoting LPPC to manage postpartum issues. However, women who experienced health issues during pregnancy were at greater risk of developing PPM. This finding corroborates previous studies on maternal near-miss cases [11, 34], which refer to instances where women survive severe, life-threatening complications during pregnancy, childbirth, or within six weeks post-delivery [35]. Ensuring effective pregnancy monitoring can therefore improve PPC uptake and help reduce maternal morbidity and mortality [36].

### What this study adds

The novel contribution of this study lies in its use of nationally representative data from Morocco to generate new insights into PPC uptake, which may also be relevant to other LMIC. The fact that only non-single women were recruited did not affected the representativeness of the data because the majority of women who deliver in Morocco are not single (in 2018, 58.5% married, 9.9% widow, 3.4 divorced, 0.6% separated) [18]. Moreover, the Moroccan law prohibits sexual relationships outside marriage, and a proof of marriage is required for delivery-related hospitalisation [37].

Moreover, the study confirms established facilitators of PPC (e.g., education, ANC visits), but also highlights system-level factors affecting care continuity. Specifically, the absence of a doctor during delivery was linked to reduced LPPC attendance, suggesting that midwife or nurse-led care in public hospitals may lack the follow-up mechanisms necessary for encouraging PPC visits.

The strong association between newborn care and maternal PPC use suggests that integrating mother–child services may be an effective strategy to boost PPC. Another key aspect explored is the temporal dynamics of care: EPPC lower the odds of later PPM and increase the odds of LPPC uptake, while the positive association between LPPC and PPM suggest that it might be used by women as a recourse in response to postpartum health symptoms. The curative perception of PPC was evidenced qualitatively in Morocco [38] and in many other LMIC [17, 39]. Therefore, implementing interventions to improve women’s health literacy is essential for a better understanding of the preventive role of PPC. Overall, these findings have practical implications for improving maternal health programs in Morocco and similar contexts.

### Limitations of this study

This study presents several limitations. The cross-sectional design precludes establishing temporality, therefore potential reverse causality notably between PPM leading to LPPC attendance or vice-versa cannot be concluded as well as causality relationships with other independent variables. Moreover, retrospective data collection may have introduced recall bias, and some independent variables including marital status, were measured at the time of the survey rather than at the time of birth. The exclusion of cases with missing data along with vulnerable subgroups such as single mothers and migrants may have resulted in selection bias. Moreover, data were self-reported without medical verification to corroborate women’s declarations, possibly leading to underreporting, particularly of psychological morbidities which were not addressed in the questionnaire. Similarly, other important factors namely cultural practices, beliefs, and women's autonomy in decision-making were not covered in the survey which may have limited the depth of insight. Additionally, the omission of these unobserved confounders in the analyses, may have resulted in residual confounding. Lastly, data lacked clarity about the number of PPC check-ups, hindering any comparison with the WHO guidelines.

## Conclusion

PPC utilisation remains low in Morocco, particularly later PPC (21.8%) while PPM persist. Both variables were marked by social health inequities with a clear social gradient based on education and household wealth index. This study showed that pregnancy monitoring encouraged continuity of care and reduced PPM occurrence. Ultimately, the findings support the need of efficient interventions promoting PPC uptake. A qualitative study may help to gather insights on the reasons that encourage women to use later PPC and their experiences and perceptions of postpartum care and morbidities.

## Supplementary Information

Supplementary Material 1.

## Data Availability

The data analysed in this study are available upon request to the Moroccan Ministry of Health- Directorate of Planning and Financial Resources, Division of Planning and Studies, Health Studies and Information Unit (Direction de la Planification et des Ressources Financières, Division de la Planification et des Etudes, Services des Etudes et de l'Information Sanitaire). Email address: dpeseis@gmail.com.

## Abbreviations

ANC: Antenatal care
AOR: Adjusted odds ratios
CI: Confidence interval
EPPC: Early postpartum care
LMIC: Low-and-middle income countries
LPPC: Later postpartum care
PNC: Postnatal care (for baby)
PPC: Postpartum care
PPM: Postpartum morbidity
WHO: World Health Organisation
