# Supplementary material for: The relationship between postpartum care uptake and postpartum morbidity and their determinants in Morocco: a secondary analysis of a national survey
Source: BMC Public Health. 2026 Apr 9;26:1623. doi: 10.1186/s12889-026-26774-x (PMC13191918; doi:10.1186/s12889-026-26774-x)
Supplement: Supplementary file 1 — Supplementary Material 1. [file 12889_2026_26774_MOESM1_ESM.pdf]

Supplementary file

Details about the three dependent variables

S. Table 1. Definitions of the dependant variables

	Question	Population
**EPPC**	328. Before being discharge from (quote the health facility declared in question 317), does someone examine you and provide you a consultation?<br>**328** — قبل ما تخرجي من (اسم المرفق الصحي المذكور في السؤال 317) واش جا شي واحد قلبك انت ودوز ليك ؟<br>Oui<br>Non<br>Ne se rappelle pas	Among the women recruited for the survey, the following inclusion criteria were applied for the women questionnaire:<br>- aged between 15 and 49 years,<br>- who gave birth to a live baby within five years prior to the survey (from 2013 to 2017)<br>- most recent live birth<br>- non-single marital status<br>Exclusion criterion:<br>- not experiencing a delivery-led hospitalisation (giving birth at home)
**LPPC**	330. During the 6 weeks after childbirth (after being discharge from the health facility declared in question 317), did you consult someone for yourself?<br>**330** — في 6 سيمانات من بعد الولادة (بعد ما خرجتي من (المرفق الصحي المذكور في السؤال 317)) واش دوزتي انت عند شي واحد ؟<br>Oui<br>Non<br>Ne se rappelle pas	Among the women recruited for the survey, the following inclusion criteria were applied for the women questionnaire:<br>- aged between 15 and 49 years,<br>- who gave birth to a live baby within five years prior to the survey (from 2013 to 2017)<br>- most recent live birth<br>- non-single marital status
**PPM**	334. During the 6 weeks after the childbirth of (name of the child), have you experienced:<br>1. acute vaginal haemorrhage,<br>2. oedema and foot pain,<br>3. smelly vaginal discharge with fever,<br>4. pelvic pain with fever, lower back pain with fever,<br>5. dorsal pain with fever, urinary burning with fever,<br>6. pain and swelling mammary with fever,<br>7. other morbidities	Among the women recruited for the survey, the following inclusion criteria were applied for the women questionnaire:<br>- aged between 15 and 49 years,<br>- who gave birth to a live baby within five years prior to the survey (from 2013 to 2017)<br>- most recent live birth<br>- non-single marital status

				Oui	Non	Ne sait pas	
		في 6 السيمانات اللي من بعد لولادة ديال (الاسم) واش :					
	334	1. جاتك الفيضة	**1.** Hémorragie vaginale aigue	1	2	8	
		2. تنفخو ليك وحرقوك رجليك	**2.** Oedème et douleurs des pieds	1	2	8	
		3. خرجات منك شي حاجة ريحتها خايبة وجاتك السخانة	**3.** Pertes vaginales malodorantes accompagnées de fièvre	1	2	8	
		4. جاك شي حريق في كرشك التحتانية و السخانة	**4.** Douleurs pelviennes accompagnées de fièvre	1	2	8	
		5. جاك شي حريق فلتحت ديال ظهرك و السخانة	**5.** Lombalgie accompagnée de fièvre	1	2	8	
		6. واش جاك شي حريق فلفوق ديال ظهرك و السخانة	**6.** Dorsalgie accompagnée de fièvre	1	2	8	
		7. جاك حريق البولة و السخانة	**7.** Brûlures mictionnelles accompagnées de fièvre	1	2	8	
		8. نفخ وحريق في بزازلك و السخانة	**8.** Douleurs et gonflement mammaire accompagnés de fièvre	1	2	8	
		9. شي حاجة اخرى ...	9. Autre(à spécifier) : ____________	1	2		

Caption: EPPC: early postpartum care, LPPC: later postpartum care, PPM: postpartum morbidity

Details about the diagnostic test conducted to select predictors included in the logistic regression analyses:

The predictor selection process was performed in four steps, which are detailed in the Table 2 (below):

Firstly, the exclusion of 23 predictors which exhibited high level of intercorrelations to avoid duplication of information and/or misrepresentation of the predictors' relative influence in the logistic regression. Secondly, the exclusion of two predictors, namely "women's partner education level" and "Person deciding for women's occupation status", due to their level of missing data, 24.8% and 51%, respectively. A possible explanation of the 24.8% of missing values for "women's partner education level" could be that this variable was coded with three modalities (i.e. primary, secondary, higher) without considering the lack of formal education. Thirdly, the exclusion of 7, 10, and 12 variables that were not significantly associated with the three outcomes respectively (i.e. Early Postpartum care (EPPC), later postpartum care (LPPC), postpartum morbidity (PPM)), in the univariate analyses. Fourthly, multicollinearity diagnostic tests were applied for each dependent variable with the remaining eligible predictors.

Due to the lack of gold standards thresholds indicating the presence of multicollinearity, cut-offs were defined based upon methodological guidance. Therefore, the following cut-off were applied to identify multicollinearity:

- If one of the variables had a variance inflation factor (VIF) higher than 2.5 (in the coefficients table) (Johnston et al., 2018).
- If one of the dimension variables had a condition index higher than 30 with a variance proportion higher than 0.5 for each variable of this dimension (in the collinearity diagnostic table) (Belsley, 1991, p.139; Draper & Smith, 1998, p.382).

Following this process, 13, 14, and 11 predictors were retained for EPPC, LPPC, and PPM, respectively. The final set of selected predictors is presented in Table 3 (below).

References

Belsley, D. A. (1991). *Conditioning diagnostics: Collinearity and weak data in regression* (Wiley).

Draper, N. R., & Smith, H. (1998). *Applied Regression Analysis*. John Wiley & Sons, Incorporated. http://ebookcentral.proquest.com/lib/uwestlon/detail.action?docID=1775203

Johnston, R., Jones, K., & Manley, D. (2018). Confounding and collinearity in regression analysis: A cautionary tale and an alternative procedure, illustrated by studies of British voting behaviour. *Quality & Quantity*, *52*(4), 1957–1976. https://doi.org/10.1007/s11135-017-0584-6

S. Table 2. Selection process of predictors analysed in the logistic regression

	Removed at step 1 (Duplicate information)	Removed at step 2 (Missing data)	Removed at step 3 (Univariate analyses)	Removed at step 4 (Multicollinearity diagnostic test)	Selected for EPPC	Selected for LPPC	Selected for PPM
- Women's age (3 categories)					X	X	X
- Women's education (3 categories)					X	X	X
- Women's partner education (3 categories)		X					
- Women's occupation status			X		X	X	
- Household wealth index					X	X	X
- Marital status			X X X				
- Place of residence					X	X	X
- regions (12 categories)			X	X X			
- regions (3 categories)	X						
- long distance from a HF preventing LPPC uptake			x NF x				
- LPPC location V1 (5 categories)			x x				X
- LPPC location V2 (3 categories)	X						
- LPPC provider V1			X X	X			
- LPPC provider V2 (dichotomous)	X						
- Reasons for not using LPPC			X	X X Linear regression did not work when this variable was included			
- ANC visit (dichotomous)	X						
- ANC visit frequency (3 categories)	X						
- ANC visit frequency (4 categories)					X	X	X
- ANC provider			X	X X			
- Mode of delivery					X	X	X
- Wanted caesarean			X	X X linear regression did not work when this variable was included			
- SBA (all types)			X		X	X	
- SBA (dichotomous)	X						
- Place of delivery (all types)					X	X	X
- HF governance			X		X	X	
- Length of stay in HF after childbirth				X X X			
- Computer usage V1 (6 categories)				X X X			
- Computer usage V2 (3 categories)	X						
- Person deciding for women's occupation status		X					
- Last ANC location V1 (5 categories)				X X			X

- Last ANC visit location V2 (public/private)	**X**						
- Morbidity during pregnancy	**X**						
- Frequency of morbidities during pregnancy			X		**X**		**X**
- PNC before discharge from HF					**X**	**X**	**X**
- PNC within 6 weeks after birth			X		**X**	**X**	
- Knowledge about breast and cervical cancers			X X	X			
- Knowledge about breast and cervical screening			X X	X			
- Experience of cervical screening			X		**X**	**X**	
- Cervical screening location			X X	X			
- Time left after the last cervical screening			X X X				
- Contraception usage			X X				**X**
- Breastfeeding			X X X				
- EPPC			**NF**			**X**	**X**
- LPPC			**NF**		**NF**		**X**
- PPM	**X**<br>not necessary as information is in PPM frequency						
- PPM frequency			X		**X**	**X**	
- Acute vaginal haemorrhage	**X**						
- Oedema and feet pain	**X**						
- Smelly vaginal discharge	**X**						
- Pelvic pain with fever			X		**X**	**X**	
- Lower back pain with fever	**X**						
- Dorsal pain with fever	**X**						
- Urinary burning with fever	**X**						
- Pain and swelling mammary with fever	**X**						
- Other symptoms related to the delivery			X X			**X**	

Caption: PPM: postpartum morbidities, LPPC: later postpartum care, PPC: postpartum care, PNC: postnatal care for baby, HF: health facility, ANC: antenatal care, SBA: skilled birth attendant, NF: not feasible

X: analysis of the associations of eligible predictors and EPPC

X: analysis of the associations of eligible predictors and LPPC

X: analysis of the associations of eligible predictors and PPM

S. Table 3. Predictors selected for the multivariate logistic regression for each outcome variable

Predictors	Categories	Dependent variables		
**Sociodemographic and environmental predictors**		**EPPC**	**LPPC**	**PPM**
Women's age	15-29/30-39/40-49	X	X	
Women's education	None/Primary/secondary and higher	X	X	X
Women's employment	Unemployed/Employed	X	X	
Socioeconomic status	Poorest/Poorer/Middle/Richer/Richest	X	X	X
Place of residence	Urban/Rural	X	X	X
**Obstetric predictors**				
ANC consultations frequency	0 /1 to 3/4/More than 4	X	X	X
Mode of delivery	Vaginal/Vaginal assisted by instrument/ Caesarean	X	X	X
Birth attendant	Doctor/Midwives or nurses/Doctors and midwives	X	X	
PPM frequency	No morbidity/1/2/3/4/5/6/7/8/9	X	X	
Occurrence of morbidities during pregnancy	No morbidity/1/2/3/4/5/6/7/8	X		X
PNC before discharge	Yes/No	X	X	X
PNC within six weeks	Yes/No	X	X	
EPPC	Yes/No		X	X
LPPC	Yes/No	X		X
Pelvic pain with fever	Yes/No		X	
Other postpartum symptoms related to the delivery	Yes/No		X	
Last ANC location	Public hospital/Public health centre/Private clinic/Private surgery/Home			X
Contraception usage	Yes/No			X

Caption: PNC: Postnatal care, ANC: antenatal care, PPM: Postpartum morbidity, LPPC: late postpartum care, EPPC: early postpartum care

S. Table 4 presents findings regarding LPPC uptake.

S. Table 4. Multilevel logistic regression of factors associated with later postpartum care uptake in Morocco

Variables	LPPC No (%)	LPPC Yes (%)	OR (95% CI)	Adjusted OR Model 1	Adjusted OR Model 2
**Early postpartum care**					
No	91.0	9.0	1		1
Yes	68.7	31.3	4.61 (3.85-5.52)***		2.68 (2.08-3.45)***
**Postpartum morbidity**					
No morbidity	79.9	20.1	1		1
1 morbidity	73.1	26.9	1.46 (1.23-1.73)***		1.36 (1.08-1.71)**
2 morbidities	73.1	26.9	1.46 (1.12-1.90)**		1.24 (0.86-1.81)
3 morbidities	76.0	24.0	1.25 (0.85-1.84)		1.14 (0.64-2.03)
4 morbidities	75.4	24.6	1.30 (0.83-2.03)		1.19 (0.59-2.40)
5 morbidities	78.0	22.0	1.12 (0.62-2.00)		0.38 (0.16-0.95)*
6 morbidities	77.9	22.1	1.12 (0.50-2.50)		0.93 (0.30-2.91)
7 morbidities	84.0	16.0	0.76 (0.25-2.30)		0.29 (0.04-2.41)
8 morbidities	61.5	38.5	2.48 (0.61-10.13)		3.20 (0.32-31.96)
**Postpartum pelvic pain with fever**					
No	78.6	21.4	1		1
Yes	73.5	26.5	1.32 (1.05-1.65)**		2.11 (1.35-3.31)**
**Other postpartum morbidities**					
No	78.9	21.1	1		1
Yes	62.4	37.6	2.25 (1.72-2.95)***		2.03 (1.38-3.00)***
**Maternal age**					
15-29	81.5	18.5	1	1	1
30-39	76.1	23.9	1.38 (1.20-1.58)***	1.42 (1.22-1.67)***	1.23 (1.02-1.48)*
40-49	75.2	24.8	1.45 (1.19-1.75)***	1.54 (1.23-1.92)***	1.17 (0.90-1.52)
**Maternal education**					
No formal education	83.6	16.4	1	1	1
Primary	74.5	25.5	1.75 (1.52-2.01)***	1.71 (1.45-2.01)***	1.34 (1.11-1.63)**
Secondary and higher	57.8	42.2	3.71 (3.05-4.52)***	2.99 (2.38-3.75)***	1.79 (1.35-2.36)***
**Partner's education level**					
Primary	82.5	17.5	1		
Preliminary/Moderate	78.9	21.1	1.26 (1.04-1.51)*		
Secondary and Higher	63.1	36.9	2.75 (2.33-3.25)**		
**Women's employment status**					
Unemployed	71.0	29.0	1	1	1
Employed	79.0	21.0	1.53 (1.26-1.86)***	1.01 (0.81-1.26)	0.81 (0.63-1.06)
**Household wealth index**					
Poorest	86.2	13.8	1	1	1
Poorer	81.6	18.4	1.40 (1.13-1.75)**	1.18 (0.91-1.53)	1.33 (0.99-1.80)
Middle	77.9	22.1	1.77 (1.43-2.18)***	1.25 (0.95-1.64)	1.25 (0.92-1.70)
Richer	74.6	25.4	2.13 (1.73-2.62)***	1.34 (1.00-1.78)*	1.42 (1.02-1.98)*

Richest	67.1	32.9	3.05 (2.47-3.78)***	1.74 (1.29-2.35)***	1.66 (1.18-2.34)**
**Place of residence**					
Rural	84.3	15.7	1	1	1
Urban	73.4	26.6	1.95 (1.70-2.23)***	1.22 (1.00-1.50)	0.97 (0.77-1.23)
**Antenatal care**					
0 visit	90.1	9.9	1		1
1 to 3 visit(s)	83.5	16.5	1.80 (1.37-2.37)***		1.64 (1.08-2.47)*
4 visits	76.7	23.3	2.77 (2.09-3.67)***		1.88 (1.23-2.86)**
> 4 visits	68.7	31.3	4.15 (3.18-5.42)***		1.89 (1.25-2.86)**
**Mode of delivery**					
Vaginal delivery	84.5	15.5	1		1
Vaginal delivery assisted by instruments	83.3	16.7	1.09 (0.92-1.30)		0.90 (0.74-1.11)
Caesarean delivery	51.7	48.3	5.10 (4.27-6.08)***		2.50 (1.89-3.31)***
**Birth attendant**					
Doctors and Nurses/Midwives	66.2	33.8	1		0.99 (0.77-1.27)
Nobody	52.5	47.5	1.77(0.77-4.11)		
Doctors	58.7	41.3	1.38 (1.13-1.68)**		1
Nurses/Midwives	86.1	13.9	0.32 (0.26-0.38)**		0.63 (0.48-0.83)***
Traditional Midwives	85.9	14.1	0.32 (0.24-0.44)**		
Relatives/Friends/ Neighbours	84.5	15.5	0.36 (0.25-0.51)**		
Another person	90.1	9.9	0.22 (0.07-0.71)*		
**Postnatal care before discharge**					
No	89.4	10.6	1		1
Yes	71.2	28.8	3.43 (2.87-4.10)***		1.15 (0.89-1.49)
**Postnatal care within 6 weeks**					
No	90.9	9.1	1		1
Yes	55.2	44.8	8.07 (6.99-9.32)***		6.97 (5.89-8.25)***
**Place of delivery**					
Home	14.9	85.1	1		
Public hospital	17.6	82.4	1.22 (0.98-1.52)		
Delivery or health centre	14.2	85.8	0.95 (0.71-1.26)		
Private clinic	49.2	50.8	5.53 (4.34-7.04)**		
Private surgery	55.3	44.7	7.05 (4.10-12.10)**		
**Length of stay in a health facility post-delivery**					
< a day (hours)	86.0	14.0	1		
1 ≥days ≤7	76.3	23.7	1.92 (1.46-2.51)**		
≥ 1 week	63.2	36.8	3.59 (1.93-6.66)**		

Caption: *: p<0.05 **: p<0.01 ***: p<0.001
Model 1: adjusted for sociodemographic and environmental variables
Model 2: adjusted for all variables
LPPC: later postpartum care
OR: odds ratios

The findings regarding PPM occurrence are displayed in S.Table 5.

S. Table 5. Multilevel logistic regression of factors associated with postpartum morbidity in Morocco

Variables	PPM No (%)	PPM Yes (%)	OR (95% CI)	Adjusted OR Model 1	Adjusted OR Model 2
**Early postpartum care**					
No	66.8	33.2	1		1
Yes	74.8	25.2	0.68 (0.60-0.77)***		0.65 (0.52-0.79)***
**Later postpartum care**					
No	73.2	26.8	1		1
Yes	66.2	33.8	1.39 (1.22-1.60)***		1.76 (1.46-2.13)***
**Maternal age**					
15-29	71.8	28.2	1		
30-39	72.2	27.8	0.98 (0.86-1.11)		
40-49	69.7	30.3	1.11 (0.93-1.32)		
**Maternal education**					
No formal education	71.4	28.6	1	1	1
Primary	70.8	29.2	1.03 (0.91-1.17)	0.95 (0.82-1.11)	0.97 (0.82-1,15)
Secondary and higher	76.3	23.7	0.78 (0.63-0.96)*	0.71 (0.56-0.91)**	0.71 (0.54-0.93)*
**Partner's education level**					
Primary	70.5	29.5	1.27 (1.07-1.49)*		
Preliminary/Moderate	72.5	27.5	1.15 (0.96-1.38)		
Secondary and Higher	75.2	24.8	1		
**Women's employment status**					
Unemployed	71.7	28.3	1		
Employed	71.5	28.5	1.01 (0.83-1.23)		
**Household wealth index**					
Poorest	73.2	26.8	1	1	1
Poorer	73.6	26.4	0.98 (0.82-1.18)	1.03 (0.80-1.31)	0.96 (0.73-1.25)
Middle	69	31	1.23 (1.03-1.47)*	1.27 (0.98-1.64)	1.16 (0.88-1.53)
Richer	69.3	30.7	1.21 (1.02-1.45)*	1.09 (0.83-1.44)	1.10 (0.81-1.48)
Richest	73.7	26.3	0.97 (0.80-1.18)	1.03 (0.77-1.38)	0.99 (0.72-1.36)
**Place of residence**					
Rural	73.3	26.7	1	1	1
Urban	70.4	29.6	1.15 (1.02-1.30)*	1.29 (1.07-1.57)**	1.17 (0.95-14.5)
**Reasons for not having received later postpartum care**					
Absence of complications	78.4	21.6	1		
Not aware of the importance of the care	67.5	32.5	1.75 (1.46-2.11)**		
High cost	44.4	55.6	4.55 (3.60-5.75)**		
Long distance	66.7	33.3	1.82 (1.23-2.67)**		
Postpartum care service not available	77.5	22.5	1.06 (0.61-1.83)		
Other	48.2	51.8	3.91 (2.58-5.93)**		
**Antenatal care**					
0 visit	69.9	30.1	1		1
1 to 3 visit(s)	72.6	27.4	0.87 (0.72-1.06)		0.30 (0.14-0.64)**
4 visits	76.7	23.3	0.71 (0.57-0.87)**		0.23 (0.11-0.50)***
> 4 visits	68	32	1.09 (0.90-1.32)		0.31 (0.14-0.66)**
**Last antenatal care location**					

Public hospital	59.0	41.0	1	1
Health centre/delivery centres	74.8	25.2	0.48 (0.37-0.63)***	0.60 (0.44-0.83)**
Private clinic	73.7	26.3	0.51 (0.35-0.76)***	0.50 (0.32-0.80)**
Private surgery	71.0	29.0	0.59 (0.46-0.76)***	0.60 (0.44-0.81)**
Home	49.3	50.7	1.48 (0.40-5.43)	1.00 (0.07-14.46)
**Morbidities during pregnancy**				
No morbidity	84.2	15.8	1	1
1 morbidity	71.0	29.0	2.17 (1.84-2.56)***	2.10 (1.72-2.56)***
2 morbidities	52.0	48.0	4.90 (4.04-5.95)***	4.21 (3.32-5.34)***
3 morbidities	47.5	52.5	5.88 (4.70-7.36)***	5.81 (4.42-7.64)***
4 morbidities	42.3	57.7	7.25 (5.53-9.50)***	6.96 (5.06-9.57)***
5 morbidities	42.2	57.8	7.28 (5.24-10.11)***	6.84 (4.59-10.18)***
6 morbidities	27.4	72.6	14.05 (8.57-23.04)***	13.17 (7.37-23.54)***
7 morbidities	27.3	72.7	14.18 (6.55-30.71)***	15.33 (5.43-43.26)***
8 morbidities	25.5	74.5	15.53 (5.31-45.37)***	7.37 (2.18-24.90)**
9 morbidities	12.2	87.8	38.39 (0.41-3612.87)	
**Postnatal care before discharge**				
No	67.0	33.0	1	1
Yes	74.2	25.8	0.71 (0.62-0.81)***	1.06 (0.86-1.31)
**Contraception usage**				
No	67.3	32.7	1	1
Yes	72.5	27.5	0.78 (0.66-0.93)*	0.88 (0.70-1.09)
**Mode of delivery**				
Vaginal delivery	74.8	25.2	1	1
Vaginal delivery assisted by instruments	70.7	29.3	1.23 (1.07-1.42)**	1.24 (1.04-1.48)*
Caesarean delivery	68.8	31.2	1.35 (1.14-1.60)**	1.15 (0.92-1.43)
**Birth attendant**				
Nobody	83.2	16.8	1	
Doctors	71.6	28.4	1.96 (0.64-5.94)	
Nurses/Midwives	73	27	1.83 (0.61-5.53)	
Doctor+ nurses/midwives	67.4	32.6	2.38 (0.78-7.26)	
Traditional Midwives	68.2	31.8	2.30 (0.75-7.06)	
Relatives/Friends/ Neighbours	74.5	25.5	1.69 (0.54-5.24)	
Another person	60.9	39.1	3.17 (0.85-11.85)	
**Place of delivery**				
Public hospital	70.6	29.4	1	
Delivery or health centre	75.3	24.7	0.79 (0.66-0.95)**	
Private clinic	72.9	27.1	0.89 (0.75-1.06)	
Private surgery	74.1	25.9	0.84 (0.47-1.49)	
Home	71.0	29.0	0.98 (0.83-1.17)	
**Length of stay in a health facility post-delivery**				
< a day (hours)	72.1	27.9	1	
1≥ days ≤7	72.1	27.9	1.00 (0.81-1.24)	
≥1 week	50.1	49.9	2.58 (1.45-4.58)**	

Caption: *: p<0.05 **: p<0.01 ***: p<0.001

Model 1: adjusted for sociodemographic and environmental variables

Model 2: adjusted for all variables

PPM: postpartum morbidity

OR: odds ratios
